# Effect of *Rigor* Stage and Pressurisation on Lipid Damage, Total Volatile Amine Formation and Autolysis Development in Palm Ruff Stored on Ice

**DOI:** 10.3390/foods12040799

**Published:** 2023-02-13

**Authors:** José M. Malga, Teresa Roco, Alfonso Silva, Gipsy Tabilo-Munizaga, Mario Pérez-Won, Santiago P. Aubourg

**Affiliations:** 1 Department of Food Technology, Instituto de Investigaciones Marinas (CSIC), 36208 Vigo, Spain; 2 Department of Food Engineering, Universidad de La Serena, La Serena 1700000, Chile; 3 Department of Aquaculture, Faculty of Marine Sciences, Universidad Católica del Norte, Coquimbo 1781421, Chile; 4 Department of Food Engineering, Universidad del Bío-Bío, Chillán 3780000, Chile

**Keywords:** *Seriolella violacea*, *rigor* stage, high-pressure processing, storage on ice, lipid hydrolysis, lipid oxidation, volatile amines, autolysis

## Abstract

The effect of the *rigor* stage (pre or post) and previous high-pressure processing (HPP; 450 and 550 MPa for 3 min) was checked during the storage on ice of farmed palm ruff (*Seriolella violacea*). Fish processed in pre-*rigor* conditions led to higher and lower levels (*p* < 0.05) of moisture and lipid contents in chilled fish, respectively, when compared to their counterpart samples processed in the post-*rigor* stage. Pre-*rigor* fish showed a higher (*p* < 0.05) quality level than post-*rigor* samples according to the assessment of the K value (59.0–92.1 and 70.3–96.3 ranges, respectively), fluorescent compounds (0.29–1.11 and 0.37–1.90 ranges, respectively), free fatty acids (FFA) (15.1–188.0 and 33.8–232.5 g·kg^−1^ lipids ranges, respectively), and total volatile amines (216.3–387.6 and 217.7–412.2 g·kg^−1^ muscle ranges, respectively). Pressure-treated fish showed higher (*p* < 0.05) quality retention than non-treated samples according to the formation of fluorescent compounds (0.29–0.86 and 0.85–1.90 ranges, respectively), FFA (15.1–50.6 and 58.9–223.5 g·kg^−1^ lipids ranges, respectively), and total volatile amines (216.3–250.3 and 351.1–412.2 g·kg^−1^ muscle ranges, respectively) and the evolution of the K value (59.0–77.2 and 86.9–96.3 ranges, respectively). The use of pre-*rigor* fish and previous HPP is recommended for the commercialisation of the current species as a fresh product.

## 1. Introduction

Marine species deteriorate rapidly post-mortem as a consequence of several degradation mechanisms such as microbial activity, autolysis, and lipid oxidation [1,2]. The increasing consumption of high-quality fresh products has required the employment of advanced technologies that guarantee sensory and safety properties while retaining nutritional value. Among such technologies, high-pressure processing (HPP) has been shown to maintain the nutritional and sensory qualities of marine species, inactivate endogenous enzyme activity and microbial development, extend their shelf-life and enhance their safety [3,4]. Notably, HPP has been successfully applied as a previous treatment to the refrigerated and chilled storage of different kinds of marine species [5].

Based on the fact that muscle degradation processes occur slowly during the pre-*rigor* stage, prolonging this physiological stage should retard flesh quality loss and increase the shelf-life time of the corresponding seafood. Therefore, previous studies have addressed the comparative effect of processing fish in both *rigor* conditions and proved the substantial advantages of employing pre-*rigor* individuals as the initial substrate [6,7,8,9]. However, most quality analyses carried out in such studies have been focused on fillet yield, physical properties, and sensory acceptance. Contrastingly, chemical changes related to quality loss, especially lipid damage, have been scarcely studied [10,11,12].

In recent decades, a notorious decrease in the availability of traditional species has occurred. Consequently, seafood manufacturers and technologists are paying increasing attention to aquaculture development as a source of food products obtained from marine fish and invertebrate species [13]. Palm ruff (*Seriolella violacea*), a pelagic fish found along the Chilean coast and the Galapagos islands, has acquired great interest as a remarkable farming product [14,15]. According to the development of this farmed species, previous research has addressed the effect of pre- and post-*rigor* conditions on quality parameters during the refrigerated (4 °C) storage of palm ruff. Such studies have concerned the thermal behaviour of proteins (FTIR spectroscopy and DSC analysis) [16,17], physical properties (water-holding capacity, firmness and ultrastructure) [18], and physico-chemical properties (volatile amines and colour parameters) [19].

As a novel study, the present research focuses on the quality loss of farmed palm ruff during storage on ice. The effect of the *rigor* stage (pre or post) of the initial fish and the effect of a previous HPP (450 and 550 MPa for 3 min) were checked during storage. To do this, different chemical indices (lipid oxidation and hydrolysis; total volatile amine formation; autolysis) related to quality loss were measured after 12 and 14 days of storage on ice. A comparison between the initial fish (both in pre- and post-*rigor* stages) and non-pressurised fish (control, 0.1 MPa) was carried out.

## 2. Materials and Methods

### 2.1. Initial Fish and Rigor Condition

Palm ruff fish were obtained from farms developed at the Catholic University of the North (Coquimbo, Chile). The length and weight of individuals ranged from 32 to 35 cm and from 675 to 700 g, respectively. Individuals were transported alive in boxes on ice to the laboratory at the University of La Serena (La Serena, Chile) (around 30 min). Then, fish were beheaded, gutted and filleted, each fillet weighting ca. 175 g. A total number of 84 fillets was prepared and kept under refrigerated (4 °C) conditions before processing.

On the same day, six fillets were taken, divided into three groups (two fillets per group) and analysed independently (*n* = 3). In each fillet, the white muscle was taken, minced, and pooled together within each group to determine the pre-*rigor* quality of the initial fish. At the same time, six fillets were subjected to refrigerated storage (4 °C for 24 h) to allow the *rigor* to set in and recede. After this time, the white muscle was taken, pooled together within each group, minced and analysed independently (*n* = 3) to determine the post-*rigor* quality of the initial fish.

The remaining 72 fillets were packed and subjected to vacuum sealing in individual high-density polyethylene bags. Bags were separated into two groups. The first group (36 fillets) was separated for carrying out the pressurisation treatment immediately after packing (i.e., pre-*rigor* group). For this, two thirds of such fillets (24 fillets) were taken for HPP, while one third (12 fillets) was directly subjected to storage on ice (pre-*rigor* control; 0.1 MPa) until analysis.

The second group (36 fillets) was refrigerated (4 °C) for 24 h to allow the *rigor* to set in and recede (i.e., post-*rigor* batch). After this time, the fillets were taken for HPP. As in the pre-*rigor* group, two thirds of such fillets (24 fillets) were subjected to HPP, while one third of the fillets (12 fillets) was directly subjected to storage on ice (post-*rigor* control; 0.1 MPa) until analysis.

In order to assess the *rigor* stage evolution of samples, pH was directly determined in the muscle with a digital pH meter (Hanna model HI 99,163, Smithfield, RI, USA).

### 2.2. HPP of Fish Samples

For HPP, vacuum-sealed fish samples were loaded in a cylindrical pressure chamber (700 mm length, 60 mm diameter) and pressurised at 450 and 550 MPa for 3 min each. Pressure was applied at room temperature (20.0 ± 2.0 °C) and measured with an integrated thermocouple in a 2 L-pilot high pressure unit (Avure Technologies, Kent, WA, USA). Water was employed as the pressure-transmitting medium. The time to reach the chosen pressure was 10 s, and decompression time was less than 5 s. An increase of 5 °C/100 MPa was detected during the pressurisation process. Since the initial fish had been kept under refrigerated conditions (4 °C) before HPP, temperature acquired by fish samples after the 450 and 550 MPa treatments was included in the 26–27 °C and 31–32 °C ranges, respectively.

For HPP, individuals corresponding to both pre- and post-*rigor* groups were divided into two groups and subjected, respectively, to 450 MPa and 550 MPa for 3 min each. Once processed, fillet samples were stored on ice until analysis.

The present HPP conditions were chosen based on previous studies carried out by our group [18,19]. In such studies, both pressure values (450 and 550 MPa) were found valuable in order to partially inhibit quality loss during the refrigeration (4 °C) of palm ruff fillets. Additionally, according to previous studies [18,19], a single pressure-holding time (i.e., 3 min) was found convenient for the present study.

### 2.3. Storage on Ice and Sampling Procedure

Sampling was carried out on the initial day (day 0; initial fish) and after 10 and 12 days of storage on ice. For each kind of sample, the white muscle of two fillets was separated and pooled together. Additionally, three different replicates (*n* = 3) were considered and analysed independently.

As for HPP conditions, sampling times (10 and 12 days) were selected based on previous research carried out by our group [18,19]. In such studies, longer storage periods were checked based on the fact that fillet samples were concerned. Since whole fish pieces were taken into account in the current study, a shorter storage period was considered to be more convenient to check possible quality differences among fish samples.

### 2.4. Determination of Moisture and Lipid Values in Palm Ruff Muscle

The moisture of initial and chilled fish muscle was assessed as the weight difference (1–2 g) before and after 4 h at 105 °C, following the official method 950.46B [20]. The resulting values were expressed as g·100 g^−1^ fish muscle.

Lipids of initial and chilled fish muscle were extracted following the Bligh and Dyer [21] method, which uses a single-phase solubilisation of the lipids employing a chloroform-methanol (1:1) mixture. Quantification of lipid extracts was carried out according to the method proposed by Herbes and Allen [22]. The resulting values were expressed as g·100 g^−1^ fish muscle.

### 2.5. Assessment of Lipid Damage Development

The conjugated diene (CD) value was assessed spectrophotometrically (Beckman Coulter DU 640 spectrophotometer, Brea, CA, USA) at 233 nm [23] and calculated according to the formula CD = A·V·w^−1^, where A is the absorbance reading and V and W are the volume (mL) and weight (mg), respectively, of the lipid extract aliquot considered for the analysis.

The peroxide value (PV) was assessed spectrophotometrically (520 nm) in the lipid extract following the method proposed by Chapman and McKay [24], in which peroxides present in the lipid extract are reduced with ferric thiocyanate. The resulting values were calculated as meq. active oxygen·kg^−1^ lipids.

The formation of fluorescent compounds (Fluorimeter LS 45; Perkin Elmer España; Tres Cantos, Madrid, Spain) was analysed in the aqueous phase resulting from the lipid extract obtained from the palm ruff white muscle. For this, measurements at 393/463 nm and 327/415 nm were carried out according to Torres et al. [25]. The relative fluorescence (RF) was calculated according to the formula: RF = *F/F_st_*, where *F* is the fluorescence measured at each excitation/emission wavelength pair and *F_st_* is the fluorescence intensity of a quinine sulphate solution (1 µg·mL^−1^ in 0.05 M H_2_SO_4_) at the corresponding wavelength pair. The results are expressed as the FR, which was calculated as the ratio between the two RF values according to the formula: FR = RF_393/463 nm_/RF_327/415 nm_.

The free fatty acid (FFA) value was assessed on the lipid extract of the fish muscle following the method proposed by Lowry and Tinsley [26]. This method is based on the formation of a complex between FFA and cupric acetate–pyridine, followed by the spectrophotometric determination at 715 nm. The resulting values were expressed as g FFA·kg^−1^ lipids.

### 2.6. Assessment of the Total Volatile Base-Nitrogen (TVB-N) Content and the K Value (%)

The TVB-N content was assessed as described previously [27]. For this, a fish muscle fraction (10 g) was extracted with 6% perchloric acid and brought up to 50 mL. TVB-N value was determined by titration of the distillate with 10 mM HCl after steam-distillation of the acid extracts rendered alkaline to pH 13 with 20% NaOH. The resulting values were expressed as mg TVB-N·kg^−1^ fish muscle.

Nucleotide extracts were obtained following the method proposed by Ryder [28]. The analysis was carried out by HPLC following the procedure proposed by Aubourg et al. [29]. The K value (%) was calculated on the basis of the following molar concentration ratio, in which the concentrations of the different molecules involved in the adenosine-triphosphate degradation pathway are taken into account: K value (%) = 100 × [hypoxanthine + inosine]/[adenosine-triphosphate + adenosine-diphosphate + adenosine-monophosphate + inosine-monophosphate + inosine + hypoxanthine].

### 2.7. Statistical Analysis

The values (*n* = 3) obtained from the different chemical determinations were subjected to the ANOVA method (*p* < 0.05) to explore differences in two different ways: the *rigor* stage (pre-*rigor* and post-*rigor* conditions) and during previous pressure treatment (Statistica version 6.0, 2001; Statsoft Inc., Tulsa, OK, USA). A comparison of means was performed using the least-squares difference (LSD) test.

## 3. Results and Discussion

### 3.1. Moisture and Lipid Contents in the Refrigerated Fish Muscle

Moisture values for initial fish and fish stored on ice ranged in all cases between 75.6 and 77.5 g·100 g^−1^ muscle values (Table 1). A definite effect on moisture content could not be observed as a result of the storage on ice. Notably, fish processed in the pre-*rigor* stage had higher average values compared to the post-*rigor* conditions. Concerning the effect of previous HPP on moisture content, a concluding trend could not be proved in fish stored for a 10-day period. However, samples corresponding to the longest storage period (i.e., 12 days) showed a decreasing tendency for average values by increasing the previous pressure applied. Thus, the lowest level (*p* < 0.05) corresponded to fish previously subjected to a 550-MPa treatment.

Initial lipid content was, in all cases, below the 1.0 g·100 g^−1^ muscle level (Table 1), which corresponds to a low-fat fish species [30,31]. Such a low-value range was maintained in fish samples throughout the storage on ice. Regarding the lipid content, all chilled fish showed lower average values in the pre-*rigor* stage compared to the counterpart from the post-*rigor* batch (Table 1). Differences showed to be significant (*p* < 0.05) in fish stored for 10 days and previously subjected to 450 and 550 MPa. Concerning the effect of previous HPP, the highest average lipid levels were detected in fish corresponding to the 450-MPa batch, differences being found significant (*p* < 0.05) in all cases except for post-*rigor* fish stored for 10 days in ice storage conditions.

According to the present results, a definite trend regarding moisture and lipid levels could not be concluded for previous HPP during subsequent chilled storage. On the other hand, processing pre-*rigor* individuals led to higher moisture levels and to lower lipid values. In the current study, values for both constituents can be explained by the influence of different and opposite degradative mechanisms. Firstly, protein denaturation during chilled storage, especially as a result of HPP, can provoke a decrease in the water holding capacity of fish muscle, leading to the release of water [3,5]. As a consequence of this decrease in moisture content, the relative content of the lipid fraction would increase in the chilled fish muscle. On the other hand, lipid hydrolysis and oxidation are likely to occur during chilled storage [1,2] and lead to lipid degradation development. Additionally, HPP has been shown to increase free metal content in muscle as a result of the denaturation of metal-bound proteins and subsequent enhancement of lipid oxidation development [3,5]. As a result of these lipid damage processes, a decrease in lipid content could be produced and lead to a relative increase in moisture level.

Previous research concerning changes in the moisture and lipid contents as a result of the *rigor* stage can be considered scarce, especially for the lipid fraction value. Thus, Le et al. [32] showed that post-*rigor* tra catfish (*Pangasius hypophthalmus*) fillets reduced the exudation loss during the chilled storage when compared to pre-*rigor* samples. However, Roco et al. [18] did not find a definite trend for moisture contents during the refrigerated storage (4 °C) of palm ruff (*S. violacea*) fillets handled under pre- and post-*rigor* conditions. Bhat et al. [3] did not observe differences in moisture content during the chilled storage of Pacific white shrimp (*Litopenaeus vannamei*) by comparing individuals in pre- and post-*rigor* conditions. Lipid content was found higher in blanched shrimp if taken in post-*rigor* conditions, but was lower in cooked shrimp in post-*rigor* when compared to their counterparts from the pre-*rigor* stage [33]. Post-*rigor* processing and increasing the refrigerated storage time (4 °C up to 12 days) led to a decrease in the moisture content in silver carp (*Hypophthalmichthys molitrix*) [34]. Bhat et al. [35] found higher moisture values in blanched and cooked Pacific white shrimp (*L. vannamei*) that were handled in pre-*rigor* conditions when compared to individuals taken in the post-*rigor* stage.

### 3.2. Lipid Oxidation Development in the Refrigerated Fish Muscle

CD content in initial fish was hardly modified in fish muscle during the chilled stage (Table 2). In most cases, average values were higher in post-*rigor* fish. However, differences were not found to be significant (*p* > 0.05). An analysis of the effect of HPP revealed an increasing tendency with the previous pressure applied in post-*rigor* fish, with differences only being significant (*p* < 0.05) in chilled fish after 10-day storage. It was concluded that both the rigor stage of the initial fish and previous pressure treatment exerted a negligible effect on the CD level.

Contrary to the CD values, all kinds of chilled fish underwent a marked peroxide formation when compared to the initial fish (Table 2). In spite of this general increase, all values remained at a score below 10, which can be considered an acceptable level for chilled seafood [2,36]. After 10-day storage, a definite effect of *rigor* condition of fish could not be proved. However, fish stored for a longer period (i.e., 12 days) revealed higher average values in post-*rigor* conditions than in their counterparts processed in the pre-*rigor* stage. Differences were found to be significant (*p* < 0.05) in samples corresponding to control and 450-MPa batches. Concerning the influence of the pressure treatment, an increasing average PV was detected in pre-*rigor* fish by increasing the pressure applied. Differences were found significant (*p* < 0.05) at day 12 between samples corresponding to control and 550-MPa batches. In contrast, a decreasing average content was observed in most cases in post-*rigor* fish by increasing the pressure applied. At day 10, differences were found significant (*p* < 0.05) between control and both pressure-treated batches.

A marked formation of fluorescent compounds was observed in chilled fish corresponding to both pre- and post-*rigor* conditions by comparison with the values obtained for the initial fish (Table 3). In fish stored for 10 and 12 days, samples chilled under pre-*rigor* conditions revealed lower average values than their counterparts from post-*rigor* conditions. Differences were found to be significant (*p* < 0.05) for palm ruff muscle corresponding to control and 450 MPa batches. Consequently, an inhibitory effect on fluorescent compound formation could be concluded by processing fish in pre-*rigor* conditions. Concerning the effect of previous HPP, a substantial decrease (*p* < 0.05) in the FR was observed by increasing the pressure applied in all kinds of samples. Therefore, a marked inhibitory effect of the pressure level could be demonstrated on the development of the fluorescent compounds in chilled muscle during the storage period under study.

Previous research focusing on the influence of HPP on the development of the lipid oxidation mechanism can be considered controversial according to the different and opposite effects that may take place. HPP can lead to a prooxidant effect as a result of a content increase in free metal ions in fish muscle resulting from metal-bound protein denaturation [3,5]. Additionally, HPP treatment has been shown to provoke remarkable modifications in the protein structure [37], so that an increased reactivity of proteins towards electrophilic compounds would be produced, therefore leading to increased fluorescent compound formation [25,38]. Furthermore, the high-pressure treatment can provoke an inhibition of the prooxidant activity of fish endogenous enzymes (lipoxygenases, peroxydases, etc.) during subsequent fish processing or storage. This effect would increase with the pressure level applied and lead to an inhibition of lipid oxidation development and, consequently, to a shelf-life increase. According to the present results on fluorescent compound formation, it can be concluded that the inhibitory effect on endogenous enzyme activity during the storage period has been the most important factor.

Previous research concerning the effect of the *rigor* stage on lipid oxidation development during subsequent storage has led to different conclusions. Most of such studies have considered a single lipid oxidation index. Thus, a higher thiobarbituric acid (TBA) value was detected in post-*rigor* silver carp (*H. molitrix*) during the refrigerated stage (12 days at 4 °C) by Jalalian et al. [34]. Furthermore, a higher primary and secondary lipid oxidation development was observed in fish corresponding to the post-*rigor* conditions during the chilled storage (14 days) of Chilean jack mackerel (*Trachurus murphyi*), although no effect was observed for the fluorescent compound formation [39]. No effect on the TBA value in refrigerated (26 days at 4 °C) palm ruff (*S. violacea*) fillets was detected by Lemus-Mondaca et al. [19] compared to pre- and post-*rigor* conditions of processing. Bhat et al. [33] did not find differences in the PV during 12-day chilled storage of Pacific white shrimp (*L. vannamei*) when comparing individuals in pre- and post-*rigor* conditions.

Concerning the effect of HPP on lipid oxidation development, previous results and conclusions provided in the literature show a great dependence on different aspects such as the analysed lipid oxidation index, the concerned type of marine species, the applied concrete HPP and the subsequent storage condition. Thus, Ohshima et al. [40] showed that the peroxide content of mackerel and cod muscle increased with the increase in previous pressure value (200–600 MPa range for 15 and 30 min). On the other hand, a partial inhibition of peroxide formation was observed in coho salmon (*Oncorhynchus kisutch*) during chilled storage for 20 days when previous HPP treatment (170 and 200 MPa for 30 s) was applied [41]. However, a substantial fluorescent compound formation was observed in a such study if coho salmon was previously subjected to a 200 MPa-30 s treatment. Vázquez et al. [42] observed a marked inhibitory influence on fluorescent compound formation during Atlantic mackerel (*Scomber scombrus*) storage (3 months at −10 °C) by increasing the pressure (150–450 MPa) and the pressure holding time (0.0–5.0 min) prior to frozen storage. In a related experiment, Torres et al. [25] proved an inhibitory effect on lipid oxidation (FR and PV assessments) during the frozen storage (3 months at −10 °C) of Atlantic jack mackerel (*Trachurus trachurus*) as a result of increasing the pressure level applied (150–450 MPa).

Inhibition of fluorescent compound formation but no effect on the peroxide level was detected in frozen (−10 °C for 5 months) hake (*Merluccius merluccius*) by prior HPP (150–450 MPa for 2 min) [43]. An inhibitory effect on the FR was evident in frozen (−10 °C) hake (*M. merluccius*) previously subjected to HPP (150 MPa for 2 min), but no effect was proved in hake stored at lower temperatures (i.e., −18 and −30 °C) [31]. Recently, Zhang et al. [12] detected an inhibitory effect on the PV, but not on the CD or the fluorescent compound formation by previous HPP (150 MPa for 5 min) in refrigerated (4 °C for 10 days) coho salmon (*O. kisutch*).

### 3.3. Lipid Hydrolysis Development in the Refrigerated Fish

A remarkable increase (*p* < 0.05) in the FFA value was proved by comparing the initial fish to any of the samples corresponding to the storage on ice (Table 3). Additionally, higher (*p* < 0.05) FFA levels were observed in fish stored for 12 days than in their counterparts stored on ice for 10 days.

Related to the effect of the *rigor* condition, higher values (*p* < 0.05) were detected in fish corresponding to the post-*rigor* stage than in their counterparts corresponding to the pre-*rigor* condition (Table 3). Therefore, an inhibitory influence on lipid hydrolysis development during subsequent chilled storage was concluded if individuals under the pre-*rigor* stage are considered for processing.

Concerning the effect of previous pressure treatment, a marked inhibitory effect (*p* < 0.05) on lipid hydrolysis development was concluded in chilled fish previously subjected to any of the high-pressure treatments tested (Table 3). Furthermore, average FFA levels of samples corresponding to the 550-MPa treatment were lower than those observed in 450-MPa conditions. Differences were found to be significant (*p* < 0.05) in fish samples stored for 10 days. A remarkable inhibitory effect of HPP was concluded on lipid hydrolysis development in fish muscle during chilled storage.

The preservative effect observed in the current study for HPP can be explained on the basis of the denaturation process of lipolytic enzymes (phospholipases and lipases in general; endogenous and microbial) and, therefore, the inhibition of their activity during subsequent storage under refrigerated conditions [44,45]. Remarkably, lipid hydrolysis is not likely to be produced by high-pressure treatment since the formation of FFA would lead to a volume increase that would not be favoured by the Le Châtelier rule [3,5]. Previous studies account for a wide number of examples showing the inhibitory influence of HPP on FFA formation during the subsequent storage of marine species under refrigerated or frozen conditions.

Previous research related to the effect of the *rigor* stage on lipid hydrolysis development can be considered scarce. A definite effect of pre/post *rigor* condition on the FFA formation could not be detected in Chilean mackerel (*T. murphyi*) during its chilled storage up to 14 days [39]. However, and according to the present results, post-*rigor* silver carp (*H. molitrix*) processing and increasing the refrigerated storage time led to an increase of the FFA value during the refrigerated (12 days at 4 °C) storage [34].

Previous research has shown the inhibitory effect of a previous HPP on the FFA formation. Thus, an inhibitory influence on lipid hydrolysis development during the chilled storage (14-day storage on ice) of Chilean jack mackerel (*T. murphyi*) was observed by Maluenda et al. [39] if a previous HPP (250, 450 and 550 MPa for 3–4 min) was applied. An inhibitory effect was concluded at the 15–20-day chilled period of coho salmon (*O. kisutch*) if previously subjected to 200 MPa for 30 s, but no influence could be proved when a lower pressure (135 or 170 for 30 s) was considered [46]. Recently, Zhang et al. [12] showed an inhibitory influence on lipid hydrolysis development in refrigerated (4 °C for 10 days) coho salmon (*O. kisutch*) by applying a prior 150 MPa/5 min treatment.

Concerning frozen seafood, previous HPP has also shown remarkable inhibition of lipid hydrolysis development. Thus, a marked inhibitory influence on FFA formation during the frozen storage (3 months at −10 °C) of Atlantic mackerel (*S. scombrus*) [42] and Atlantic jack mackerel (*T. trachurus*) [25] was observed as a result of the increase in pressure level and the pressure holding time (150–450 MPa for 0.0–5.0 min) prior to frozen storage. A decreased FFA formation in frozen hake (*M. merluccius*) was concluded by previous HPP (150–450 MPa for 2 min) prior to frozen storage (−10 °C for 5 months) [43]. Recently, Carrera et al. [31] proved an inhibitory effect on the lipid hydrolysis development in frozen hake (*M. merluccius*) kept at −10 and −18 °C by previous HPP (150 MPa for 2 min), although such an effect could not be demonstrated in hake stored at −30 °C.

### 3.4. TVB-N Content in the Refrigerated Fish

Comparison with the initial fish showed that all kinds of refrigerated samples underwent a great formation of total volatile amines (Table 4). After 10-day storage, TVB-N values were slightly higher in pre-*rigor* conditions when compared to their post-*rigor* counterparts. However, significant differences (*p* > 0.05) were not detected. In contrast, palm ruff stored for 12 days showed higher average values in the case of samples processed in the post-*rigor* stage. Differences were found to be significant (*p* < 0.05) in 450- and 550-MPa batches. Consequently, a concluding trend for the effect of *rigor* conditions on the initial fish could not be demonstrated on the formation of total volatile amines during the storage period under consideration.

Concerning the effect of the pressure treatment, lower (*p* < 0.05) TVB-N values were obtained in palm ruff previously subjected to pressure processing when compared to the control. In most cases, lower average values were obtained in samples corresponding to the 550-MPa batch when compared to their counterparts from the 450-MPa treatment. Differences were found to be significant (*p* < 0.05) in pre-*rigor* fish stored for 12 days in ice. Therefore, an inhibitory influence of pressure treatment on TVB-N value was concluded.

Food proteins have been shown to be modified and damaged by spoilage bacteria and endogenous enzymes, this giving rise to the formation of nitrogen-containing volatile amines [1,2]. Thus, an increase in the TVB-N level during the *rigor* period has been explained on the basis of the bacterial metabolism [10]. Furthermore, a TVB-N increase has been reported to be facilitated by HPP during storage on ice as a result of protein denaturation [3,5].

Previous research has already addressed the influence of the fish *rigor* stage on the formation of volatile amines. Thus, a higher TVB-N value was detected during the refrigerated storage (6–8 °C) of post-*rigor* rainbow trout (*Oncorhynchus mykiss*) and mirror carp (*Cyprinus carpio*) [10]. Jalalian et al. [34] showed that post-*rigor* processing and refrigeration time led to an increase in the TVB-N value during the refrigerated storage (12 days at 4 °C) of silver carp (*H. molitrix*). A higher formation of TVB-N was observed in fish corresponding to the post-*rigor* condition during the storage on ice (14 days) of Chilean jack mackerel (*T. murphyi*) [39]. Additionally, higher trimethylamine (TMA)-N and TVB-N values were obtained in post-*rigor* Pacific white shrimp (*L. vannamei*) than in pre-*rigor* samples subjected to blanching and cooking [35]. Contrary to such results, Bhat et al. [33] did not find differences in TVB-N value during the chilled storage of Pacific white shrimp (*L. vannamei*) by comparing individuals in pre- and post-*rigor* conditions.

According to the present results, previous investigation has shown an inhibitory effect of previous HPP on volatile amine formation during a subsequent storage of seafood. Remarkably, studies have been addressed to single amines such as TMA and dimethylamine (DMA). Thus, a previous HPP (300 MPa fopr 20 min) effectively reduced the TMA and DMA content and the shelf-life time was extended in refrigerated (4 °C) squid (*Todarodes pacificus*) [47]. Later on, Malinowska-Pańczyk and Kołodziejska [48] proved that the formation of TMA in refrigerated (4 °C) cod (*Gadus morhua*) muscle could be strongly reduced if previously subjected to a high-pressure treatment (193 MPa at −20 °C). Furthermore, inhibition of TMA and DMA formation in frozen (−10 °C for 5 months) hake (*M. merluccius*) was detected by Vázquez et al. [43] by previous HPP (150–450 MPa for 2 min). Recently [31], a previous HPP (150 MPa for 2 min) of hake (*M. merluccius*) led to the inhibitory formation of TMA and DMA during subsequent storage under frozen conditions (−10 and −18 °C).

### 3.5. K Value (%) of the Fish Muscle

The autolysis development in chilled palm ruff muscle was determined by the K value (%). Compared to the initial palm ruff, a great increase in this index was proved in all kinds of chilled samples (Table 4). Furthermore, a marked increase was detected in fish muscle by increasing the chilling time from 10 to 12 days. An effect of increasing the chilling time on the K value has already been reported in seafood in general [2,49].

Lower average K values were observed in samples corresponding to the pre-*rigor* condition when compared to their counterparts from the post-*rigor* stage. Differences were found to be significant (*p* < 0.05) in fish chilled for 10 (450- and 550-MPa batches) and 12 (control batch) days. Therefore, an inhibitory influence on autolysis assessment was concluded by processing palm ruff in the pre-*rigor* stage.

Related to the effect of previous pressure processing, the highest (*p* < 0.05) K values were observed in chilled fish corresponding to the control batch. Additionally, lower average values were obtained in samples corresponding to the 550-MPa batch when compared to their counterparts from the 450-MPa treatment. Differences were found significant (*p* < 0.05) in pre-*rigor* fish after 10-day chilled storage. Consequently, a substantial effect on the K value could be proved for previous pressure treatment, this effect leading to partial inhibition of this autolysis mechanism.

Previous research has shown a higher retention of ATP value in pre-rigor fish than in their counterpart in post-*rigor* conditions [50,51]. However, negligible information is available concerning the effect of *rigor* conditions on the K value. On the other hand, several previous studies account for the influence of HPP on the K value of refrigerated seafood. In most cases, and according to the present results, a K-value decrease resulting from inactivation of enzymes that are able to catalyse the adenosine 5′-triphosphate (ATP) degradation by previous HPP has been proposed. This research concerns different kinds of seafood such as tilapia (*Oreochromis niloticus*) fillets (50–300 MPa for 0–60 min) [52], yellowfin tuna (*Thunnus albacares*) chunks (100, 200 and 300 MPa for 5 min) [53] and white prawn (*Fenneropenaeus indicus*) (100, 270, 435 and 600 MPa for 5 min) [54]. A reduction in enzyme activity was detected in such studies, although inactivation was not complete. Zhang et al. [55] subjected squid (*T. pacificus*) to HPP (200–600 MPa) and observed a hypoxanthine content decrease during subsequent refrigerated storage (10 days at 4 °C), this effect increasing with the pressure applied. In contrast, no effect on the K value was observed by Vázquez et al. [43] in frozen (−10 °C for 5 months) hake (*M. merluccius*) if previously subjected to HPP (150–450 MPa for 2 min).

## 4. Conclusions

The marked effect of *rigor* conditions of the initial fish and previous pressure treatment was detected on the quality changes in farmed palm ruff during storage on ice. Fish processed in pre-*rigor* conditions led to higher and lower levels (*p* < 0.05) of moisture and lipid contents, respectively, in chilled fish when compared to their counterpart samples processed in the post-*rigor* stage. Pre-*rigor* fish showed a higher quality level than post-*rigor* samples according to the assessment of the K value, fluorescent compounds, FFA, and TVB-N. Pressure-treated fish showed a higher quality retention than non-treated samples according to the formation of fluorescent compounds, FFA, and total volatile amines and to the evolution of the K value.

This study provides valuable information concerning the chemical changes related to quality loss of a remarkable farming species during its storage on ice. Such information would complement previous knowledge concerning quality changes related to sensory, physical, and microbiological properties during the refrigerated storage (4 °C) of palm ruff. According to the current results, the use of initial fish in pre-*rigor* conditions and the employment of a previous HPP are strongly recommended for the commercialisation of the current fish species as a fresh product. Based on the current depletion of most wild fish species and the great interest shown by the fish trade in the development of profitable farmed species, further research on palm ruff ought to be carried out. In such further studies, the effect of internal (size, sex, maturity degree, fat content) and external (diet, slaughtering season) factors on quality loss ought to be taken into account.

## Figures and Tables

**Table 1:** Determination (g·100 g^−1^ muscle) * of the moisture and lipid contents in chilled palm ruff corresponding to different initial *rigor* conditions and previous high-pressure processing (HPP) **.

Chemical Constituent	Chilling Time (days)	*Rigor* Condition (pre/post)	Previous HPP (MPa)		
			0.1 (Control)	450	550
Moisture content	Initial	Pre	76.94 a (0.05)		
		Post	77.37 b (0.33)		
	10	Pre	77.48 bC (0.10)	76.15 bA (0.08)	76.69 aB (0.02)
		Post	75.60 aA (0.02)	75.82 aA (0.15)	76.62 aB (0.01)
	12	Pre	77.42 bB (0.08)	77.34 bB (0.05)	76.88 bA (0.09)
		Post	76.89 aC (0.03)	76.32 aB (0.15)	75.77 aA (0.05)
Lipid content	Initial	Pre	0.81 b (0.04)		
		Post	0.68 a (0.03)		
	10	Pre	5.3 aA (0.3)	6.8 aB (0.8)	5.0 aA (0.2)
		Post	6.2 aA (0.5)	9.9 bB (0.7)	9.1 bB (1.1)
	12	Pre	5.8 aA (0.5)	8.7 aB (0.2)	6.5 aA (0.1)
		Post	6.0 aA (0.3)	10.5 bB (1.2)	6.8 aA (0.1)

* Average values of three replicates (*n* = 3); standard deviations are indicated in brackets. ** Different lowercase letters (a,b) indicate significant (*p* < 0.05) differences as a result of the *rigor* stage. Different capital letters (A,B,C) indicate significant (*p* < 0.05) differences as a result of previous HPP.

**Table 2:** Lipid oxidation determination * in chilled palm ruff corresponding to different initial *rigor* conditions and previous high-pressure processing (HPP) **.

Quality Index	Chilling Time (days)	*Rigor* Condition (pre/post)	Previous HPP (MPa)		
			0.1 (Control)	450	550
Conjugated diene (CD) formation ***	Initial	Pre	0.81 a (0.14)		
		Post	0.73 a (0.12)		
	10	Pre	0.81 aA (0.09)	0.81 aA (0.20)	0.83 aA (0.08)
		Post	0.59 aA (0.21)	0.82 aAB (0.09)	1.01 aB (0.09)
	12	Pre	0.74 aA (0.08)	0.68 aA (0.21)	0.72 aA (0.11)
		Post	0.79 aA (0.12)	0.89 aA (0.09)	1.07 aA (0.12)
Peroxide value (PV) (meq. active oxygen·kg^−1^ lipids)	Initial	Pre	2.81 b (0.94)		
		Post	0.96 a (0.55)		
	10	Pre	4.65 aA (0.62)	5.16 aA (0.44)	5.62 aA (1.34)
		Post	5.89 aB (0.38)	4.81 aA (0.05)	4.11 aA (0.44)
	12	Pre	5.58 bA (0.06)	6.49 aAB (1.26)	6.81 aB (0.40)
		Post	7.96 bA (0.41)	8.31 bA (0.10)	6.84 aA (1.96)

* Average values of three replicates (*n* = 3); standard deviations are indicated in brackets. ** Different lowercase letters (a,b) indicate significant (*p* < 0.05) differences as a result of the *rigor* stage. Different capital letters (A,B) indicate significant (*p* < 0.05) differences as a result of previous HPP. *** CD units were calculated as the following ratio [23]: absorbance reading x volume (mL) of the aliquot sample/weight of the aliquot sample (mg).

**Table 3:** Determination * of fluorescence ratio (FR) and free fatty acid (FFA) content in chilled palm ruff corresponding to different initial *rigor* conditions and previous high-pressure processing (HPP) **.

Quality Index	Chilling Time (days)	*Rigor* Condition (pre/post)	Previous HPP (MPa)		
			0.1 (Control)	450	550
FR	Initial	Pre	0.42 a (0.26)		
		Post	0.89 a (0.11)		
	10	Pre	0.85 aC (0.03)	0.40 aB (0.02)	0.29 aA (0.03)
		Post	1.50 bC (0.19)	0.61 bB (0.02)	0.37 aA (0.03)
	12	Pre	1.11 aC (0.03)	0.54 aB (0.08)	0.40 aA (0.03)
		Post	1.90 bC (0.45)	0.86 bB (0.04)	0.53 aA (0.20)
FFA content (g·kg^−1^ lipids)	Initial	Pre	43.6 a (5.2)		
		Post	45.1 a (4.8)		
	10	Pre	58.9 aC (3.8)	22.4 aB (0.6)	15.1 aA (3.5)
		Post	118.4 bB (10.4)	39.0 bA (2.8)	33.8 bA (6.1)
	12	Pre	188.0 aB (6.3)	35.2 aA (2.9)	30.3 aA (6.3)
		Post	232.5 bB (2.3)	50.6 bA (5.0)	47.1 bA (4.8)

* Average values of three replicates (*n* = 3); standard deviations are indicated in brackets. ** Different lowercase letters (a,b) indicate significant (*p* < 0.05) differences as a result of the *rigor* stage. Different capital letters (A,B,C) indicate significant (*p* < 0.05) differences as a result of previous HPP.

**Table 4:** Determination * of total volatile base-nitrogen (TVB-N) content and K value (%) in chilled palm ruff corresponding to different initial *rigor* conditions and previous high-pressure processing (HPP) **.

Quality Index	Chilling Time (days)	*Rigor* Condition (pre/post)	Previous HPP (MPa)		
			0.1 (Control)	450	550
TVB-N content (g·kg^−1^ muscle)	Initial	Pre	161.3 a (11.6)		
		Post	162.7 a (7.4)		
	10	Pre	357.2 aB (5.4)	219.5 aA (4.7)	227.3 aA (6.9)
		Post	351.1 aB (37.5)	218.3 aA (9.0)	217.7 aA (5.3)
	12	Pre	387.6 aC (27.5)	230.3 aB (4.5)	216.3 aA (0.5)
		Post	412.2 aB (7.2)	250.3 bA (13.3)	243.0 bA (9.1)
K value (%)	Initial	Pre	14.4 ± 1.9 a		
		Post	15.01 ± 1.2 a		
	10	Pre	86.9 aC (2.5)	65.3 aB (1.3)	59.0 aA (1.2)
		Post	90.1 aB (1.3)	71.1 bA (1.2)	70.3 bA (1.2)
	12	Pre	92.1 aB (1.1)	75.2 aA (1.4)	73.7 aA (1.4)
		Post	96.3 bB (1.5)	77.2 aA (1.1)	76.1 aA (1.1)

* Average values of three replicates (*n* = 3); standard deviations are indicated in brackets. ** Different lowercase letters (a,b) indicate significant (*p* < 0.05) differences as a result of the *rigor* stage. Different capital letters (A,B,C) indicate significant (*p* < 0.05) differences as a result of previous HPP.

## Data Availability

Data is contained within the article.
